# High-Power, Short-Duration Ablation under the Guidance of Relatively Low Ablation Index Values for Paroxysmal Atrial Fibrillation: Long-Term Outcomes and Characteristics of Recurrent Atrial Arrhythmias

**DOI:** 10.3390/jcm12030971

**Published:** 2023-01-27

**Authors:** Shuyu Jin, Weidong Lin, Xianhong Fang, Hongtao Liao, Xianzhang Zhan, Lu Fu, Junrong Jiang, Xingdong Ye, Huiyi Liu, Yanlin Chen, Sijia Pu, Shulin Wu, Hai Deng, Yumei Xue

**Affiliations:** 1 The Second School of Clinical Medicine, Southern Medical University, Guangzhou 510515, China; 2 Guangdong Provincial People’s Hospital (Guangdong Academy of Medical Sciences), Southern Medical University, Guangzhou 510080, China; 3 School of Medicine, South China University of Technology, Guangzhou 510006, China

**Keywords:** paroxysmal atrial fibrillation, high-power, short-duration, radiofrequency ablation, ablation index, pulmonary vein reconnection, non-pulmonary vein triggers

## Abstract

Objective: The purpose of this study was to evaluate the difference in effectiveness and safety of high-power, short-duration (HPSD) radiofrequency catheter ablation (RFA) guided by relatively low ablation index (AI) values and conventional RFA in paroxysmal atrial fibrillation (PAF) patients. Methods: The HPSD RFA strategy (40–50 W, AI 350–400 for anterior, 320–350 for posterior wall; *n* = 547) was compared with the conventional RFA strategy (25–40 W, without AI; *n* = 396) in PAF patients who underwent their first ablation. Propensity-score matching analyses were used to compare the outcomes of the two groups while controlling for confounders. Results: After using propensity-score matching analysis, the HPSD group showed a higher early recurrence rate (22.727% vs. 13.636%, *p* = 0.003), similar late recurrence rate, and comparable safety (*p* = 0.604) compared with the conventional group. For late recurrent atrial arrhythmia types, the rate of regular atrial tachycardia was significantly higher in the HPSD group (*p* = 0.013). Additionally, the rate of chronic pulmonary vein reconnection and non-pulmonary vein triggers during repeat procedures was similar in both groups. Conclusions: For PAF patients, compared with the conventional RFA strategy, the HPSD RFA strategy at relatively low AI settings had a higher early recurrence rate, similar long-term success rate, and comparable safety.

## 1. Introduction

With global population aging and chronic disease survival rates increasing, both the incidence and prevalence of atrial fibrillation (AF) show a tendency of deterioration [1]. In the 2020 European Society of Cardiology (ESC) guidelines, catheter ablation to improve symptoms of AF recurrences was given a Class I recommendation for AF refractory to medical therapy [2]. Pulmonary vein isolation (PVI), acting as the cornerstone of AF radiofrequency catheter ablation (RFA) [3,4], aims to produce continuous, transmural, and durable lesions around the pulmonary veins (PVs). The ablation index (AI) is a novel marker of lesion quality during RFA that is strongly correlated with lesion depth, width, and volume [5]. Compared with conventional applications (20–35 W at the posterior wall, 35–40 W in the other segments, 10–30 s), the high-power, short-duration (HPSD) ablation strategy is characterized by higher radiofrequency power (≥40 W) and shorter duration (5–15 s). In vitro and in vivo models demonstrated that the HPSD RFA strategy made transmural lesions broader and shallower, with fewer steam pops than the conventional RFA strategy at proper settings [6]. Multiple studies have been conducted to investigate the efficacy and safety of the HPSD strategy. However, whether the HPSD strategy is more effective than the conventional strategy is still debated. Compared to the conventional strategy, Kewcharoen J et al. [7] reported that the HPSD strategy was not associated with increased freedom from atrial tachyarrhythmia at the 12-month follow-up, but other studies [8,9] showed that the HPSD strategy had higher freedom from atrial arrhythmia. Pulmonary vein reconnection (PVR) and non-PV triggers could be the dominant mechanism of paroxysmal AF (PAF) recurrence [10,11]. Furthermore, age, gender, left atrial (LA) size, deterioration of the left ventricular diastolic dysfunction, posterior wall isolation, and CHA_2_DS_2_-VASc scores have been found to be independent risk factors associated with AF recurrence after RFA [12,13,14]. Meanwhile, animal studies indicate that the HPSD settings can create more durable lesions [15,16], which may reduce the rate of chronic PVR. However, little data regarding chronic PVR patterns and non-PV triggers in repeat procedures have been published comparing the HPSD and conventional RFA settings. Therefore, in this study, we sought to compare the effectiveness and safety of the HPSD RFA settings guided by relatively low AI values with the conventional RFA settings for PAF. In addition, we evaluated the sites of chronic PVR and non-PV triggers in patients of both groups who underwent repeat ablation in order to provide further guidance for RFA.

## 2. Methods

### 2.1. Study Population

In this single-center prospective cohort study, we investigated 1176 PAF patients who underwent RFA at Guangdong Provincial People’s Hospital from July 2019 to March 2021 and followed them prospectively. All the patients signed a written informed consent form for ablation procedures. All the participants met the following criteria: (1) included patients’ age ≥ 18; (2) patients with PAF refractory to medical therapy and undergoing initial catheter ablation. The exclusion criteria were as follows: (1) previous cardiac surgeries or/and AF ablations; (2) a history of rheumatic valvular disease and ischemic heart disease; (3) LA diameter > 55 mm; (4) patients who failed to complete the procedure due to complications.

All the ablation procedures were performed by nine doctors with over 300 ablation experiences each. Anti-arrhythmia drugs (AADs) were stopped five half-lives prior to ablation. All the patients were required to uninterruptedly take non-vitamin K oral anticoagulants (NOACs) for at least 3 weeks prior to the ablation procedure. LA thrombus was excluded by transesophageal echocardiography or PV computed tomography (CT) within 72 h before the procedure. NOACs were suspended once on the morning of the procedure and recovered 4 h after the procedure.

### 2.2. Ethics Statement

The registry was approved by the ethics committee of Guangdong Provincial People’s Hospital (No. GDREC2019568H, approved on 23 September 2019) and local institutional review boards. Written informed consent was obtained from all individual participants included in the study.

### 2.3. Ablation Procedure

All the patients underwent RFA under conscious sedation with fentanyl. Under local anesthesia, the right femoral vein was punctured three times, a 7F vascular sheath was put in place, and two 8.5F SL1 vascular sheaths were delivered. A diagnostic decapolar catheter (Triguy™ Steerable Decapolar Mapping Catheter; APT Medical, Shenzhen, China) was placed in the coronary sinus. Intravenous heparin was continuously administered to maintain an activated coagulation time (ACT) at 300 to 350 s during the procedure. After two successful transseptal punctures, three-dimensional mapping of the LA was obtained using a multielectrode catheter (PentaRay Nav Catheter or Lasso; Biosense-Webster Inc., Diamond Bar, CA, USA) with the guidance of the CARTO three-dimensional mapping system (Biosense Webster Inc., Diamond Bar, CA, USA). Then, an open-irrigated, 3.5 mm cooled-tip catheter (Thermocool SMART TOUCH^®^ Uni-Directional Catheter or Thermocool SMART TOUCH^®^ SF Uni-Directional Navigation Catheter; Biosense Webster Inc., Diamond Bar, CA, USA) was used for ablation. All accepted bilateral PVI, non-PV triggers, and additional linear ablations were performed at the operators’ discretion. If there was an absence of isolation, touch-up ablation was performed until complete PVI was achieved. Ibutilide or/and electrical cardioversion was/were administered when the AF rhythm remained unconverted during ablation.

In the HPSD group guided by AI, PVI was performed at 40–50 W targeting AI values of 350–400 in the anterior, 350–380 at the superior and inferior wall of the PV, and 320–350 at the posterior wall of the LA with a CF of 5–15 g, irrigation flow rate of 15–30 mL/min per site (ST catheter at 30 mL/min; STSF catheter at 15 mL/min; specific sites adjusted according to operators’ experience), and an inter-lesion distance (ILD) of 4 mm. For additional ablation, in the HPSD group, the output power of the mitral isthmus and posterior wall isolation were 40–50 W with AI values of 350–400, that of the tricuspid isthmus was 35 W, and that of the coronary sinus was 25 W. While in the conventional group without the guidance of AI, the power setting of PVs was limited to 35 W at the anterior wall, 25–35 W at the posterior wall, and 35–40 W in the other segments. RF applications did not last more than 30 s, and every single RF delivery was performed with 10 to 20 g CF.

For recurrent patients who underwent repeat ablation procedures, the appropriate three-dimensional mapping system was selected according to the type of recurrent arrhythmia, which included an Ensite Velocity system (Abbott, St. Paul, MN, USA), a Rhythmia™ system (Boston Scientific, Marlborough, MA, USA), and a CARTO system.

### 2.4. Follow-Up

Patients were evaluated at 1, 3, and 6 months, followed by 6-month intervals up to one year, by electrocardiogram (ECG) or 24 h Holter and when symptoms were reported. Atrial arrhythmia recurrence was defined as any symptomatic or asymptomatic atrial arrhythmia lasting > 30 s after ablation. Recurrence within 3 months after the first ablation was defined as early recurrence, and recurrence after 3 months was defined as late recurrence. For patients suffering from recurrent atrial arrhythmias, repeat procedures were considered after 3 months.

Oral anticoagulants were continued for 3 months after ablation procedure unless uncontrolled bleeding or invasive procedures appeared. Long-term use of anticoagulants depended on CHA_2_DS_2_-VASc scores. AADs were appropriately selected according to operators’ discretion after ablation. After excluding the contraindications, AADs (amiodarone or propafenone) were routinely used for 3 months postoperatively, and the decision to continue AADs was based on the patient’s Holter results and symptoms 3 months after the procedure. If recurrence occurred during follow-up, the addition of AADs was recommended, and if the AF load was heavy and symptoms were severe, a repeat ablation was recommended.

### 2.5. Statistical Analysis

Continuous variables are presented as the mean ± standard deviation (x ± s). Data were analyzed using Student’s *t* test for two-group comparisons if normally distributed or the Mann–Whitney U test for non-parametric two-group comparisons. Categorical variables are presented as frequencies and percentages (%), which were analyzed using a chi-squared test or Fisher exact test between two groups. We performed propensity-score matching using the nearest neighbor method without a replacement and a caliper at a 1:1 ratio of the two groups. The variables age, LA diameter, LVEF, left ventricular diastolic dimension, CHA_2_DS_2_-VASc scores, and ablation strategy were included in the propensity-score matching. The standardized mean differences of all adjusted variables were under 0.02 after propensity-score matching. The time-to-arrhythmia recurrence was estimated using the Kaplan–Meier method and compared using the log-rank test. A two-sided *p*-value < 0.05 was considered statistically significant. A multivariable Cox proportional hazards regression analysis was used to investigate any predictors associated with one-year AF recurrence. Multifactorial analysis of survival data was performed using Cox regression if the assumption of equal proportional risk was satisfied. If the assumption of equal proportional risk was not satisfied, a non-equal proportional Cox regression analysis was considered to study the effect of prognostic factors. The variables with *p* ≤ 0.2 in the univariate Cox regression analysis and age were included in the multivariate Cox regression analysis. Statistical analyses were performed using SPSS 26.0 (IBM, Armonk, NY, USA).

## 3. Results

### 3.1. Baseline Characteristics of Patients

A total of 1176 PAF patients who underwent their first ablation from July 2019 to March 2021 were included, of whom 29 (2.466%) were lost to follow-up. A total of 943 patients completed at least the one-year follow-up, of whom 547 patients were in the HPSD RFA strategy group and 396 received the conventional RFA strategy. In the HPSD group and conventional group, the average age was 60.005 ± 10.977 and 60.333 ± 11.216 years, with 63.620% and 60.606% of patients being male, respectively. The baseline characteristics were not significantly different between the two groups except LA diameter (*p* = 0.002) and left ventricular diastolic dimension (*p* = 0.042) (Table 1).

### 3.2. Procedural Results

PVI was achieved in all patients. Additionally, the ablation strategies were different between the two groups. Superior vena cava isolation was performed in 88 (16.088%) and 58 (14.646%) patients in the HPSD and conventional groups, respectively. The proportion of mitral isthmus ablation (7.130% vs. 1.768%, *p* < 0.001) and epicardial ablation in the coronary sinus (3.473% vs. 0.505%, *p* = 0.002) was significantly higher in the HPSD group than in the conventional group. The proportions of intraoperative use of ibutilide (10.603% vs. 19.192%, *p* < 0.001) and electrical cardioversion (3.839% vs. 8.838%, *p* < 0.001) were higher in the conventional group (Table 2).

After using propensity-score matching analysis, new subsets (HPSD and conventional group, *n* = 308 each) were obtained. No significant differences were observed between the two groups with regard to baseline characteristics and performed procedures (Supplementary materials, Tables S1 and S2).

### 3.3. Follow-Up Outcomes

The follow-up durations differed between the two groups, so we evaluated the atrial arrhythmia recurrence within 12 months after a single ablation. Propensity-score matching analysis showed that the early recurrence rate in the HPSD group was still significantly higher than in the conventional group (22.727% vs. 13.636%, *p* = 0.003). For early recurrent atrial arrhythmia types, 54.286% had AF in the HPSD group compared with 52.381% in the conventional group (*p* = 0.845). Additionally, there was no statistically significant difference in the late recurrence rate after a single procedure between two groups (19.805% vs. 15.584%, *p* = 0.170). However, for late recurrent atrial arrhythmia types, regular atrial tachycardia was more common in the HPSD group compared with the conventional group (65.574% vs. 41.667%, *p* = 0.013) (Table 3).

**Figure 1:**
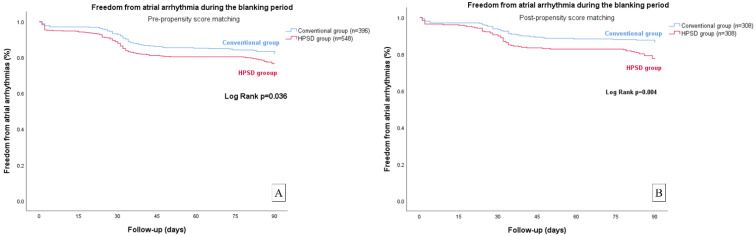
Kaplan–Meier survival analysis of freedom from arrhythmia during the blanking period. (**A**) Freedom from arrhythmia during the blanking period before propensity-score matching; (**B**) Freedom from arrhythmia during the blanking period after propensity-score matching.

**Figure 2:**
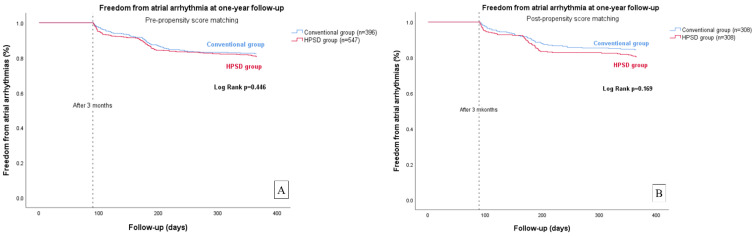
Kaplan–Meier survival analysis of freedom from arrhythmia at one-year follow-up. (**A**) Freedom from arrhythmia at one-year follow-up before propensity-score matching; (**B**) Freedom from arrhythmia at one-year follow-up after propensity-score matching.

In the Kaplan–Meier survival analysis, the early recurrence rate was higher in the HPSD group than in the conventional group (log rank, *p* = 0.036 before propensity score matching; *p* = 0.004 after propensity score matching) (Figure 1). Additionally, the rate of freedom from atrial arrhythmia at one year after a single procedure was similar between the two groups (Log rank, *p* = 0.446 before propensity score matching; *p* = 0.169 after propensity score matching) (Figure 2). In the multivariate Cox regression analysis, after adjusting for important covariates including sex, age, and LA diameter, LA diameter was associated with late recurrence within a year (hazard ratio (HR) 1.046, 95% confidence interval (CI) 1.016–1.077, *p* = 0.003) (Table 4).

### 3.4. Findings in Repeat Procedures

During 11.19 ± 0.82 months follow-up, a total of 41 patients underwent repeat procedures, including one patient who underwent repeat ablation for atrioventricular nodal reentry tachycardia (AVNRT) and another for LA appendage closure due to high CHA_2_DS_2_-VASc scores. Eventually, 15 patients in the HPSD group and 24 patients in the conventional group were analyzed. In the conventional group, one patient underwent a third procedure for recurrent regular atrial tachycardia. Superior vena cava isolation was performed in 2 (13.333%) and 5 (20.833%) patients in the HPSD and conventional groups, respectively. Additionally, the proportion of cavotricuspid isthmus ablation was higher in the conventional group than in the HPSD group (6.667% vs. 50.000%, *p* = 0.006). The baseline characteristics and first procedure characteristics of the recurrent patients are shown in Table 5 and Table 6.

**Figure 3:**
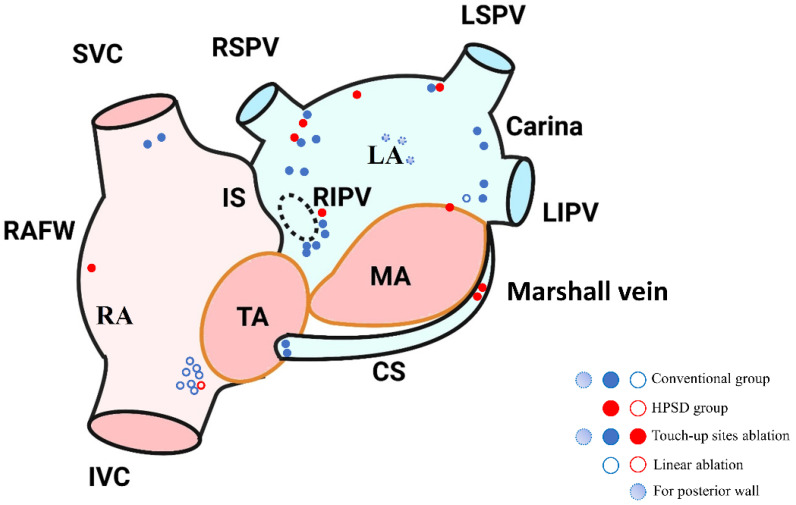
Anteroposterior schematic diagram of trigger sites during repeat procedure for recurrent patients.

The spatial distribution of chronic PVR differed between the two groups. In the HPSD group, chronic PVR occurred in 9 out of 15 (60.000%) patients, including 5 left superior PVs, 4 left inferior PVs, 7 right superior PVs, and 5 right inferior PVs. In the conventional group, 18 out of 24 (75.000%) patients had chronic PVR during redo procedures, with 9 left superior PVs, 8 left inferior PVs, 13 right superior PVs, and 12 right inferior PVs. There was no significant difference in the rate of chronic PVR between the two groups (*p* = 0.478). Additionally, the two groups had similar rates of non-PV triggers (40.000% vs. 29.167%, *p* = 0.361). In terms of PV triggers, there were 5/15 (33.333%) recurrences with a total of 5 sites in the HPSD group and 8/24 (33.333%) recurrences with a total of 15 sites in the conventional group. Meanwhile, for non-PV triggers, there were 6/15 (40.000%) recurrences with a total of 6 sites in the HPSD group and 7/24 (29.167%) recurrences with a total of 13 sites in the conventional group (Table 7). The non-PV trigger sites were located in the Marshall vein, the epicardial surface of the LA roof, the cavotricuspid isthmus, the mitral isthmus, the right atrial free wall, the mitral annulus, the superior vena cava, and the coronary sinus. The specific trigger sites are detailed in Figure 3 and Supplementary Materials Table S3.

The dots indicate the site of the trigger. The red dots represent the HPSD group, and the blue dots represent the conventional group.

### 3.5. Safety Outcomes

As is shown in Table 8, no significant difference was observed in the incidence rate of complications between two groups (*p* = 0.604). During the perioperative period, in the HPSD group, five patients had pericardial effusion, of which one patient had a reduction in pericardial effusion after conservative treatment, three patients received insertion of a pericardial drain, and one patient required surgical treatment. In the conventional group, three patients who developed pericardial effusion required insertion of a pericardial drain. During the follow-up period, no patients in either group had thromboembolic events or strokes. However, two patients died in the conventional group, one due to cancer progression and the other was unclear.

## 4. Discussion

### 4.1. Main Findings

This single-center, prospective cohort study demonstrated that the HPSD RFA strategy guided by relatively low AI values had a higher early recurrence rate, a similar late recurrence rate, and comparable safety when compared with the conventional strategy in PAF patients during the one-year follow-up. For recurrent atrial arrhythmia types, the rate of early recurrence types was similar in both groups, but the rate of recurrence with regular atrial tachycardia for late recurrent patients was significantly higher in the HPSD group according to propensity-score matching. Furthermore, no significant difference was found in the rate of chronic PVR and non-PV triggers during repeat procedures in both groups.

### 4.2. The Success Rate with Different Application Settings

The success rate of AF ablation has risen over the past two decades with the evolution of three-dimensional electroanatomic mapping, contact force sensing catheters, and catheter irrigation [17]. Cryoablation is a promising alternative technique to RFA for treating PAF with encouraging results. Chen YH et al. [18] demonstrated that cryoablation presented comparable long-term AF/atrial tachycardial-free survival and procedure-related adverse events compared with RFA. Meanwhile, cryoablation markedly shortens the procedure time with negligible impact on the fluoroscopy time. However, the HPSD ablation strategies are only applicable when using single point-by-point ablation devices. AI and lesion size index (LSI) are novel ablation quality markers to predict lesion quality that incorporates CF, time, and power in a weighted formula [19]. However, the exact power settings and AI values that result in a high success rate with AF are unclear. Currently, the setting of foreign and domestic mainstream AI is 400–600 for the anterior wall and 400–450 for the posterior wall. However, the results vary from study to study, with similar or slightly higher success rates in the HPSD group with the guidance of AI values than in the conventional group [20,21,22,23]. With the targeted AI values of 450–500 for the anterior wall and 350–400 for the posterior wall in two groups, Liu Z et al. demonstrated the HPSD-AI group (≥45 W) had lower recurrence of atrial arrhythmia at 12 months (6.8% vs. 28.3%, *p* = 0.011), higher PV first-pass isolation, shorter ablation times, and fewer patients with anatomical leakages and sites of unreached AI compared with the low power-AI group (<35 W) [24]. O’Brien J et al. [23] illustrated that with a power setting of 50 W and target AI values of 550–600 in the anterior LA region and 400–450 in the posterior LA region, there was no significant difference in the success rate at 12-month follow-up compared with the AI-guided conventional group with a power setting of 35–40 W (80.2% vs. 82.8%, *p* = 0.918). Similarly, for PAF patients, there was no significant difference in the success rate between the HPSD group (40 W) and the conventional group (30 W) at 12 months of follow-up under the same AI-guided ablation (400 for posterior and 500 for anterior wall) in both groups (92% vs. 84%, respectively, *p* = 0.22) [25]. In addition, the measurement of the local impedance might predict optimal lesion formation. A local impedance drop more than 21.80 ohms on the anterior wall and more than 18.30 ohms on the posterior wall significantly increased the probability of creating a successful lesion [26]. Boussoussou M et al. [27] found that LA wall thickness did not influence the first-pass isolation rate during PVI guided by the modified CLOSE protocol (AI 400 on the posterior wall and 500 on the anterior wall, CF 10–40 g). Only the diameter of the right superior PV was associated with the success rate of right-sided first pass isolation, as a wider right superior PV diameter led to an easier first-pass isolation.

However, fewer studies have compared the effectiveness of a HPSD group with a conventional group guided by relatively low AI values. Solimene F et al. [28] reported on 156 AF patients (124 PAF patients and 32 PeAF patients) undergoing AI-guided PVI with target AI values of 400–450 for anterior and 330–350 for posterior LA regions, with 89.2% of patients (91% PAF vs. 78% PeAF) being in the sinus rhythm at 14 ± 6 months. Okamatsu H et al. [29] investigated 60 AF patients undergoing AI-guided PVI (AI values of 400 at the anterior, 360 at the posterior LA wall, and 260 on the esophagus) randomly assigned to 3 groups (LP group: 30 W at the anterior and 20 W at the posterior wall; MP group: 40 W at the anterior and 30 W at the posterior wall; HP group: 50 W at the anterior, 40 W at the posterior wall and 30 W on the esophagus) and found no significant difference among the groups (100%, 95%, and 95% in LP, MP, and HP groups, respectively, *p* = 0.44).

Additionally, a very high power ablation strategy applied clinically improved procedural efficiency with comparable safety compared with the conventional strategy. Kottmaier M et al. [30] demonstrated that very high power, short-duration (vHPSD) applications with a high power of 70 W for 5–7 s had significantly less arrhythmia recurrence during the one-year follow-up (26.9% vs. 34.9%, *p* < 0.013) with no major complications. Additionally, vHPSD ablation performed by applying 90 W for 4 s during follow-up had more than a 90% success rate with comparable safety [31,32]. A prospective, observational cohort study showed that both HPSD (50 W, AI 500 on the anterior and 400 on the posterior LA wall) and vHPSD RFA settings (90 W/4 s) shorten procedure and RF time and result in a higher rate of first-pass isolation at 9-month AF recurrence rate (10%, 8% and 36%, *p <* 0.01) compared to LPLD RFA settings (30 W, AI 500 on the anterior and 400 on the posterior LA wall). Moreover, the presence of first-pass isolation was associated with a lower AF recurrence rate at 9 months (OR = 0.09, 95% CI 0.04–0.24, *p <* 0.01) [33].

In our study, after using propensity-score matching, compared with the conventional RFA strategy (without AI, 35 W at anterior wall, 25–35 W at the posterior wall, and 35–40 W was applied in the other segments), the HPSD RFA strategy with relatively lower AI values (40–50 W, AI values of 350–400 for anterior, 350–380 for superior and inferior, and 320–350 for posterior LA wall) had higher early recurrence, similar freedom from atrial arrhythmia, and comparable safety during 12 months of follow-up. Additionally, we are a high-volume electrophysiology center with operators who have over 10 years of AF ablation experience, and there may be no significant difference between the guidance with AI and non-AI ablation. AI is a quantitative metric that may help shorten the learning curve. We used a relatively low AI-guided ablation, which may reflect better long-term outcomes if ablation guided with a relatively high AI is currently available at other centers.

The internal aspect of the LA is relatively smooth, but its wall thickness is not uniform, with an average thickness of 4.5 ± 0.6 mm (range, 3.5–6.5 mm) at the roof, 3.9 ± 0.7 mm (range, 2.5–4.9 mm) at the left lateral wall, and 3.3 ± 1.2 mm (range, 1.5–4.8 mm) at the anterior wall, 2 mm near the vestibule of the mitral annulus, and 4.1 ± 0.7 mm (range, 2.5–5.3 mm) at the posterior wall. The wall thickness of PVs varied from heart to heart. At 0.5 cm away from the junction, the thickness of the left superior PV (LSPV), left inferior PV (LIPV), right superior PV (RSPV), and right inferior PV (RIPV) was 2.8 ± 0.5 mm (range, 1.9–3.5 mm), 1.5 ± 0.4 mm (range, 0.9–2.1 mm), 2.5 ± 0.5 mm (range, 1.8–3.3 mm) and 2.0 ± 0.4 mm (range, 1.5–2.5 mm), respectively. At 0.5 cm away from the junction, the thickness of the LSPV, LIPV, RSPV, and RIPV was 2.3 ± 0.4 mm (range, 1.8–2.8 mm), 1.2 ± 0.3 mm (range, 0.5–1.7 mm), 2.0 ± 0.3 mm (range, 1.5–2.5 mm), and 1.5 ± 0.2 mm (range, 0.9–2.2 mm), respectively. Additionally, the LA posterior wall thickness increased from the most superior to the most inferior measured level, whereas the wall was thinner in the middle and between the inferior venous orifices in those with AF (for AF patients: 2.1 ± 0.9 mm between the superior PVs, 2.2 ± 1.0 mm in the center of the posterior LA wall, and 2.5 ± 1.3 mm between the inferior PVs; for non-AF patients: 2.3 ± 1.0 mm between the superior PVs, 2.6 ± 1.0 mm in the center of the posterior LA wall, and 2.9 ± 1.3 mm between the inferior PVs) [34,35,36]. The myocardium of the left lateral ridge at its superior level was thicker than at its inferior level (2.8 ± 1.1 (range 1.5–4.2 mm) vs. 1.7 ± 0.8 mm (range 0.5–3.5 mm), respectively, *p* < 0.001) [37]. The minimal distance between the right PV antrum, left PV antrum, LA posterior wall, and esophageal wall was 6.3 ± 2.8 mm (range, 3.4–11.5 mm), 5.6 ± 2.2 mm (range, 3.3–10.5 mm), and 6.2 ± 2.5 mm (range, 3.6–13.5 mm), respectively [38]. The HPSD RFA strategy with a power of 50 W for 7 s (LSI 4.8 ± 0.52) creates wider but shallower lesions that had a diameter of 4.98 ± 0.91 mm and a depth of 2.2 ± 0.76 mm, whereas the conventional RFA strategy with a power of 25 W for 30 s (LSI 4.73 ± 0.59) had a diameter of 4.45 ± 0.74 mm and a depth of 2.8 ± 1.56 mm [39]. Additionally, the HPSD settings for 90 W/4 s in the beating heart of swine produced wider lesions (6.02 ± 0.2 mm vs. 4.43 ± 1.0 mm) and similar depth (3.58 ± 0.3 mm vs. 3.53 ± 0.6 mm) compared with the conventional RFA strategy for 25 W/20 s (CF of two groups range from 5–40 g) [16]. From the anatomy of the LA, the thickness of the posterior wall ranged from 2.5 mm to 5.3 mm [36]. Therefore, using relatively conservative AI values in patients is more consistent with safety principles, while AI values of 320–350 for posterior walls are able to balance effectiveness and safety.

### 4.3. Characteristics of Recurrent Atrial Arrhythmia

In a recent publication by Vassallo F et al. [40], the HPSD RFA strategy increased the incidence of early recurrence and reduced late recurrence compared with the conventional strategy, while AT or AFL occurring during the blanking period is more common in the HPSD group. However, Bunch et al. [41] reported a similar recurrence of AF at one year and three years between two groups, and a higher rate of AFL at one year and three years was observed in patients treated with the HPSD RFA. In our study, the propensity-score matching analysis showed that a similar recurrence of AF was observed during the blanking period in both groups, while a higher rate of atrial arrhythmia was observed after one year’s follow-up in patients treated with the HPSD RFA strategy.

The early recurrence for AF patients was associated with an inflammatory process caused by the 40–50 W power setting used in this ablation protocol [42]. After the blanking period, the spontaneous resolution of the arrhythmias with sinus rhythm conversion may be explained by the decrease in inflammatory responses or the use of AADs. Because the HPSD RFA strategy had relatively low AI values, shallower lesions, and less total energy, transmural damage may not be achieved, resulting in no difference in the late recurrence rate between the two groups. Therefore, further studies are required to explore the most optimal power setting and AI values for RFA to yield greater clinical value.

PVI is the cornerstone of AF ablation, whereas PVR is attributed to catheter instability, tissue edema, and reversible non-transmural injury [43]. Therefore, the continuous and transmural lines are key to the success of ablation. In animal studies, HPSD applications resulted in 100% contiguous lines with all transmural and improved lesions showing lesion-to-lesion consistency compared with conventional applications [15,16]. Recent studies [44,45,46,47] have shown different consequences in terms of chronic PVR during the second procedure between two groups. Yavin HD et al. [47] demonstrated that incidence of chronic PVR during the redo procedure was lower in a HPSD group than in a conventional group (16.66% vs. 52.2%, *p* = 0.03), and reconnection sites occurred in the septal aspect of the right PV or the anterior left PV in the HPSD group and in the anterior left PV, septal right PV, and posterior wall in the conventional group. However, Hansom SP et al. [45] reported that the right PV carinal segments had a higher rate of reconnection in HPSD applications compared with conventional applications, but there was no difference in chronic PVR between the two groups.

In our study, there was no difference in the proportion of chronic PVR during repeat procedure between the two groups. Some patients who did not undergo redo procedures had recurrence during the follow-up period. Additionally, areas of chronic PVR were co-located at sites of decreased catheter stability in both groups. Reconnection sites occurred more in right PVs and left carina in both groups. In terms of anatomy, it is more difficult for a catheter to reach right PVs than left PVs. Furthermore, the myocardial thickness of the carina is thicker, and the catheter attachment of the carina is more difficult, and thus the HPSD strategy with conservative AI values and lower energy may lead to non-transmural lesions. Therefore, for all the reasons listed above, we may underestimate the rate of chronic PVR, contributing to chronic PVR in both groups. Non-PV triggers were frequently found in the superior vena cava, LA anterior wall, LA posterior wall, coronary sinus, and vein of Marshall [48]. In our study, since 15% of patients in both groups underwent superior vena cava isolation in the first ablation procedure, the number of patients requiring superior vena cava isolation in the repeat ablation procedure was minimal. Additionally, both groups had similar rates of PV triggers. The proportion of non-PV triggers was relatively high in the HPSD group compared with the conventional group, but there was no significant difference in non-PV triggers between the two groups.

This implies that the relatively low AI values might not have produced transmural injury lesions, while the thicker carina and the difficulty of catheter apposition during the procedure contributed to the chronic PVR in both groups. Furthermore, a limited number of recurrent patients had repeat procedures, and additional lesions were performed during the initial procedure in both groups, which might have reduced the incidence of non-PV triggers. More well-designed and large-scale RCTs are required to confirm these findings.

### 4.4. Safety Outcomes

Safety during ablation RFA is of worthwhile concern. The primary reason for tissue injury during RFA is thermally mediated, including resistive and conductive heating. It causes irreversible damage to the myocardium once a temperature of approximately 50 °C has been reached [49]. Contrary to the conventional ablation strategy, the HPSD ablation strategy results in higher resistive heating, lower conductive heating, larger lesion diameters, and smaller lesion depths, which may reduce collateral injury to surrounding structures such as the esophagus [16,50]. HPSD ablation with a power setting of 50 W for 6 s will not result in severe esophageal temperature increases when lesions are >20 mm away from a temperature sensor [51]. Via intraoperative esophageal temperature monitoring or late gadolinium enhancement (LGE) magnetic resonance imaging (MRI) within 24 h post-ablation, it can be seen that the two groups have similar esophageal temperature dynamics and comparable esophageal tissue injury results [52,53]. In addition to the incidence of esophageal injury, the overall complications were very low in the HPSD application performed at 45–50 W, with one death due to an atrioesophageal fistula and 33 cases of cardiac tamponade in 10,284 patients [54]. Similar to other findings, the HPSD RFA strategy with relatively low AI values appears to be as safe as the conventional RFA strategy. Additionally, combined with previous anatomical data on the LA, the thickness of the posterior wall ranges from 2.5 mm to 5.3 mm, and AI values of 320–350 in the posterior wall of the LA may have a positive effect on the prevention of atrioesophageal fistula.

## 5. Limitations

There are several limitations to our study. First, this study is a single-center study. Additionally, there were variations in the ablation parameters and ablation strategies compared with other centers. Thus, the results need to be further validated by clinical randomized prospective studies. In addition, limited by way of the follow-up, the onset time may not be recorded in time for patients with asymptomatic recurrence, which may underestimate the rate of freedom from atrial arrhythmia. Additionally, not all patients with recurrence underwent electrophysiological examination and a repeat ablation, which may bias the proportion of chronic PVR and non-PV triggers during repeat procedures. Finally, in our study, we lacked data on procedure time, ablation time, and fluoroscopy time, resulting in an inability to compare efficacy between the two groups in many respects. We still need a multicenter, prospective randomized controlled study with uniform ablation strategy to further validate our findings.

## 6. Conclusions

Compared with the conventional group, the HPSD group with relatively low AI settings had a higher early recurrence rate and a similar late recurrence rate with comparable safety. Additionally, whereas both groups exhibited a similar recurrence rate of AF throughout the blanking period, patients treated with the HPSD RFA strategy experienced a higher rate of atrial arrhythmia after the one-year follow-up. Meanwhile, the rate of chronic PVR and non-PV triggers was similar between the two groups. The HPSD RFA strategy with relatively low AI settings was demonstrated to be a feasible, effective, and safe approach to PAF ablation.

## Figures and Tables

**Table 1:** Baseline characteristics of the study patients.

Variable	HPSD Group (*n* = 547) (%)	Conventional Group (*n* = 396) (%)	*p* Value
Age (year)	60.005 ± 10.977	60.333 ± 11.216	0.654
Male	348 (63.620)	240 (60.606)	0.346
Hypertension	222 (40.585)	161 (40.657)	0.982
Diabetes	76 (13.894)	57 (14.394)	0.828
Stroke	53 (9.689)	39 (9.848)	0.935
Peripheral vascular disease	32 (5.850)	29 (7.323)	0.364
Heart failure (LVEF ≤ 50%)	13 (2.377)	16 (4.040)	0.144
CHA_2_DS_2_-VASc scores	2.013 ± 1.767	2.154 ± 1.685	0.217
Smoke	70 (12.797)	47 (11.869)	0.670
Prior PCI	42 (7.678)	31 (7.828)	0.932
LA diameter (mm)	35.901 ± 4.867	36.949 ± 5.387	0.002
LVEF (%)	64.244 ± 6.185	64.108 ± 6.498	0.749
LVDD (mm)	45.266 ± 4.308	45.887 ± 4.842	0.042
AF duration [months, M (P_25_, P_75_)] *	12.000 (5.000–36.000)	12.000 (3.000–48.000)	0.750
Pacemaker implantation during follow-up	2 (0.366)	2 (0.505)	1.000
LAAC	24 (4.388)	16 (4.040)	0.794

AF: atrial fibrillation; CHA_2_DS_2_-VASc = congestive heart failure, hypertension, age ≥ 75 years, diabetes mellitus, stroke, vascular disease, age 65–74 years, sex category (female); HPSD: high-power, short-duration; LVEF: left ventricular ejection fraction; LVDD: left ventricular diastolic dimension; LAAC: left atrial appendage closure; LA: left atrial; PCI: percutaneous coronary intervention; *: results are presented as median (interquartile range).

**Table 2:** Procedure characteristics.

Variable	HPSD Group (*n* = 547) (%)	Conventional Group (*n* = 396) (%)	*p* Value
Performed procedure			
SVC isolation	88 (16.088)	58 (14.646)	0.546
LA roof line ablation	61 (11.152)	54 (13.636)	0.250
LA inferior line ablation	16 (2.925)	13 (3.283)	0.627
LA anterior wall line ablation	4 (0.731)	2 (0.505)	0.987
CTI ablation	165 (30.165)	143 (36.111)	0.055
MI ablation	39 (7.130)	7 (1.768)	<0.001
Endocardial ablation in coronary sinus	16 (2.925)	9 (2.273)	0.538
Epicardial ablation in coronary sinus	19 (3.473)	2 (0.505)	0.002
CFAE ablation	8 (1.463)	3 (0.758)	0.492
Intraoperative use of ibutilide	58 (10.603)	76 (19.192)	<0.001
Intraoperative conversion to sinus rhythm with ibutilide	37/58 (63.793)	52/76 (68.421)	0.574
Intraoperative use of electrical cardioversion	21 (3.839)	35 (8.838)	<0.001
Intraoperative conversion to sinus rhythm with electrical cardioversion	21/21 (100.000)	35/35 (100.000)	1.000

CFAE: complex fractionated atrial electrograms; CTI: cavotricuspid isthmus; HPSD: high-power, short-duration; LA: left atrial; MI: mitral isthmus; SVC: superior vena cava.

**Table 3:** Characteristics of recurrent atrial arrhythmias between two groups after a single procedure.

Variable	Pre-Propensity Score Matching			Post-Propensity Score Matching		
	HPSD Group (*n* = 547) (%)	Conventional Group (*n* = 396) (%)	*p* Value	HPSD Group (*n* = 308) (%)	Conventional Group (*n* = 308) (%)	*p* Value
Early recurrence	129 (23.583)	72 (18.182)	0.046	70 (22.727)	42 (13.636)	0.003
AF	81 (62.791)	37 (51.389)	0.115	38 (54.286)	22 (52.381)	0.845
Atrial tachycardia	48 (37.209)	35 (48.611)		32 (45.714)	20 (47.619)	
Late recurrence	107 (19.561)	70 (17.677)	0.465	61 (19.805)	48 (15.584)	0.170
AF	39 (36.449)	34 (48.571)	0.109	21 (34.426)	28 (58.333)	0.013
Atrial tachycardia	68 (63.551)	36 (51.429)		40 (65.574)	20 (41.667)	

AF: atrial fibrillation; HPSD: high-power, short-duration.

**Table 4:** Cox regression analysis for one-year atrial arrhythmia recurrence.

Variable	Univariate		Multivariate	
	HR (95% CI)	*p* Value	HR (95% CI)	*p* Value
Age (year)	1.000 (0.987–1.014)	0.952	0.996 (0.982–1.010)	0.576
Male	0.798 (0.592–1.076)	0.139	0.736 (0.539–1.006)	0.055
Hypertension	0.892 (0.658–1.208)	0.459		
Stroke	1.069 (0.843–1.356)	0.582		
Peripheral vascular disease	1.047 (0.583–1.881)	0.879		
Heart failure (LVEF ≤ 50%)	0.470 (0.066–3.353)	0.451		
CHA_2_DS_2_-VASc scores	0.978 (0.897–1.066)	0.612		
Smoke	0.834 (0.518–1.343)	0.456		
Prior PCI	0.949 (0.540–1.669)	0.856		
LA diameter (mm)	1.043 (1.013–1.073)	0.005	1.046 (1.016–1.077)	0.003
LVEF (%)	1.007 (0.983–1.032)	0.579		
LVDD (mm)	0.997 (0.964–1.030)	0.848		
AF duration (months)	0.999 (0.996–1.003)	0.774		
HPSD ablation	0.999 (0.996–1.003)	0.774		

AF: atrial fibrillation; CHA_2_DS_2_-VASc = congestive heart failure, hypertension, age ≥ 75 years, diabetes mellitus, stroke, vascular disease, age 65–74 years, sex category (female); CI: confidence intervals; HPSD: high-power, short-duration; HR: hazard ratio; LVEF: left ventricular ejection fraction; LVDD: left ventricular diastolic dimension; LA: left atrial; PCI: percutaneous coronary intervention.

**Table 5:** Baseline characteristics of patients undergoing repeat procedures.

Variable	HPSD Group (*n* = 15) (%)	Conventional Group (*n* = 24) (%)	*p* Value
Age (year)	54.867 ± 9.702	61.125 ± 11.521	0.088
Male	8 (53.333)	12 (50.000)	1.000
Hypertension	3 (20.000)	13 (54.167)	0.049
Diabetes	1 (6.667)	3 (12.500)	1.000
Stroke	0 (0.000)	3 (12.500)	0.271
Peripheral vascular disease	0 (0.000)	4 (16.667)	0.146
Heart failure (LVEF ≤ 40%)	0 (0.000)	0 (0.000)	1.000
CHA_2_DS_2_-VASc scores	1.333 ± 1.447	2.625 ±1.789	0.024
Smoke	2 (13.333)	3 (12.500)	1.000
LA diameter (mm)	34.286 ± 3.221	37.833 ± 5.939	0.047
LVEF (%)	63.286 ± 3.950	65.042 ± 3.962	0.195
LVDD (mm)	45.143 ± 3.634	46.000 ± 4.294	0.535
AF duration [months, M (P_25_, P_75_)] *	12 (6.000–72.000)	15 (3.500–57.000)	0.898

AF: atrial fibrillation; HPSD: high-power, short-duration; LVEF: left ventricular ejection fraction; LVDD; left ventricular diastolic dimension; LA: left atrial; *: results are presented as median (interquartile range).

**Table 6:** First procedure characteristics of patients undergoing repeat procedures.

Variable	HPSD Group (*n* = 15) (%)	Conventional Group (*n* = 24) (%)	*p* Value
Performed procedure			
SVC isolation	2 (13.333)	5 (20.833)	0.686
LA roof line ablation	1 (6.667)	3 (12.500)	1.000
LA inferior line ablation	0 (0.000)	0 (0.000)	1.000
LA anterior wall line ablation	1 (6.667)	0 (0.000)	0.385
CTI ablation	1 (6.667)	12 (50.000)	0.006
MI ablation	0 (0.000)	1 (4.167)	1.000
Endocardial ablation in coronary sinus	0 (0.000)	0 (0.000)	1.000
Epicardial ablation in coronary sinus	1 (6.667)	0 (0.000)	0.385
CFAE ablation	0 (0.000)	1 (4.167)	1.000
Intraoperative use of ibutilide	2 (13.333)	6 (25.000)	0.450
Intraoperative conversion to sinus rhythm with ibutilide	1/2 (50.000)	5/6 (83.333)	0.464
Intraoperative use of electrical cardioversion	1 (6.667)	2 (8.333)	1.000
Intraoperative conversion to sinus rhythm with electrical cardioversion	1/1 (100.00)	2/2 (100.00)	1.000

CFAE: complex fractionated atrial electrograms; CTI: cavotricuspid isthmus; HPSD: high-power, short-duration; LA: left atrial; MI: mitral isthmus; SVC: superior vena cava.

**Table 7:** Repeat procedure characteristics of recurrent patients.

Variable	HPSD Group (*n* = 15) (%)	Conventional Group (*n* = 24) (%)	*p* Value
**Recurrent arrhythmia type**			0.907
PAF	7 (46.667)	12 (50.000)	
PeAF	1 (6.667)	2 (8.333)	
Atrial tachycardia	6 (40.000)	7 (29.167)	
AF + Atrial tachycardia	1 (6.667)	3 (12.500)	
**PVR during redo procedure**	9 (60.000)	18 (75.000)	0.478
Left superior PVR	5 (33.333)	9 (37.500)	1.000
Left inferior PVR	4 (26.667)	8 (33.333)	0.734
Right superior PVR	7 (46.667)	13 (54.167)	0.748
Right inferior PVR	5 (33.333)	12 (50.000)	0.343
Left carina	1 (6.667)	4 (16.667)	0.631
Right carina	1 (6.667)	5 (20.833)	0.376
**PV triggers**	5 (33.333)	8 (33.333)	1.000
**Non-PV triggers**	6 (40.000)	7 (29.167)	0.361

AF: atrial fibrillation; HPSD: high-power, short-duration; PV: pulmonary vein; PVR: pulmonary vein reconnection; PAF: paroxysmal atrial fibrillation; PeAF: persistent atrial fibrillation.

**Table 8:** Complications in the two groups.

Variable	HPSD Group (*n* = 547) (%)	Conventional Group (*n* = 396) (%)	*p* Value
Total complication events	5 (0.914)	5 (1.263)	0.604
During the perioperative period			
Pericardial effusion	5 (0.914)	3 (0.758)	1.000
During the follow-up period			
Strokes	0 (0.000)	0 (0.000)	1.000
Thromboembolic Events	0 (0.000)	0 (0.000)	1.000
Death *	0 (0.000)	2 (0.505)	0.176

HPSD: high-power, short-duration; *: two patients died in the conventional group, one due to cancer progression and the other unknown.

## Data Availability

The data presented in this study are available on request from the corresponding author.

## Supplementary Materials

The following supporting information can be downloaded at: https://www.mdpi.com/article/10.3390/jcm12030971/s1; Table S1: Baseline characteristics of the study patients after propensity-score matching; Table S2: Procedure characteristics after propensity-score matching; Table S3: The specific trigger sites in both groups.
