# Supplementary material for: High-Power, Short-Duration Ablation under the Guidance of Relatively Low Ablation Index Values for Paroxysmal Atrial Fibrillation: Long-Term Outcomes and Characteristics of Recurrent Atrial Arrhythmias
Source: J Clin Med. 2023 Jan 27;12(3):971. doi: 10.3390/jcm12030971 (PMC9917927; doi:10.3390/jcm12030971)
Supplement: Supplementary file 1 [file jcm-12-00971-s001.zip › jcm-2105711-supplementary.pdf]

Supplementary materials

Table S1 Baseline characteristics of the study patients after propensity-score matching.

Variable	HPSD group (n=308) (%)	Conventional group（n=308）(%)	P value
Age (y)	59.961 ± 11.627	60.188 ± 11.248	0.805
Male	194 (62.987)	187 (60.714)	0.561
Hypertension	134 (43.506)	122 (39.610)	0.327
Diabetes	39 (12.662)	38 (12.338)	0.903
Stroke	33 (10.714)	26 (8.442)	0.338
Peripheral vascular disease	20 (6.494)	25 (8.117)	0.439
Heart failure (LVEF≤50%)	8 (2.597)	10 (3.247)	0.632
CHA_2DS_2-VASc scores	2.10 ± 1.80	2.09 ± 1.65	0.963
Smoke	44 (14.286)	38 (12.338)	0.477
Prior PCI	24 (7.792)	25 (8.117)	0.882
LA diameter (mm)	36.219 ± 4.772	36.208 ± 5.183	0.977
LVEF (%)	64.672 ± 6.174	64.549 ± 6.178	0.804
LVDD (mm)	45.649 ± 4.413	45.737 ± 4.816	0.813
AF duration [months, M (P_{25}, P_{75})] *	12.000 (6.000-36.000)	12.000 (3.000-48.000)	0.839
Pacemaker implantation during follow-up	3 (0.974)	2 (0.649)	1.000
LAAC	24 (7.792)	16 (5.195)	0.191

AF: atrial fibrillation; CHA_2DS_2-VASc = congestive heart failure, hypertension, age >_75 years, diabetes mellitus, stroke, vascular disease, age 65 - 74 years, sex category (female); HPSD: high-power, short-duration; LVEF: left ventricular ejection fraction; LVDD; left ventricular diastolic dimension; LA: left atrial; *: results are presented as median (interquartile range).

Table S2 Procedure characteristics after propensity-score matching.

Variable	HPSD group (n=308) (%)	Conventional group（n=308）(%)	P value
Performed procedure			
SVC isolation	46 (14.935)	47 (15.260)	0.910
LA roof line ablation	14 (4.545)	15 (4.870)	0.849
LA inferior line ablation	7 (2.273)	4 (1.300)	0.545
LA anterior wall line ablation	0 (0.000)	0 (0.000)	1.000
CTI ablation	100 (32.468)	100 (32.468)	1.000
MI ablation	7 (2.273)	5 (1.623)	0.560
Endocardial ablation in coronary sinus	6 (1.948)	4 (1.300)	0.752
Epicardial ablation in coronary sinus	5 (1.623)	2 (0.649)	0.450
CFAE ablation	2 (0.649)	2 (0.649)	1.000
Intraoperative use of ibutilide	30 (9.740)	31 (10.065)	0.893

Intraoperative conversion to sinus rhythm with ibutilide	8/30 (26.667)	8/31 (25.806)	0.939
Intraoperative use of electrical cardioversion	12 (3.896)	15 (4.870)	0.555
Intraoperative conversion to sinus rhythm with electrical cardioversion	12/12 (100.000)	15/15 (100.000)	1.000

CFAE: complex fractionated atrial electrograms; CTI: cavotricuspid isthmus; HPSD: high-power, short-duration; LA: left atrial; MI: mitral isthmus; SVC: superior vena cava.

Table S3 The specific trigger sites in both groups.

**Trigger sites**	**HPSD group (n=15)**	**Conventional group (n=24)**
RSPV	2	3
RIPV	1	5
LSPV	1	1
LIPV	1	2
Right carina	0	2
Left carina	0	2
RAFW	1	0
Marshall vein	2	0
Epicardial surface of LA roof	1	0
CS	0	0
CTI	1	7
MI	0	1
Mitral annulus at one o'clock	1	0
LAPW	0	3
SVC	0	2
No trigger focus recorded	3	4

CS: coronary sinus; CTI: cavotricuspid isthmus; LA: left atrium; LAPW: left atrial posterior wall; LIPV: left inferior pulmonary vein; LSPV: left superior pulmonary vein; MI: mitral isthmus; RA: right atrium; RAFW: right atrial free wall; RIPV: right inferior pulmonary vein; RSPV: right posterior pulmonary vein; SVC: superior vena cava
